# Integration of Residue Attributes for Sequence Diversity Characterization of Terpenoid Enzymes

**DOI:** 10.1155/2014/753428

**Published:** 2014-05-11

**Authors:** Nelson Kibinge, Shun Ikeda, Naoaki Ono, Md. Altaf-Ul-Amin, Shigehiko Kanaya

**Affiliations:** Graduate School of Information Science, Nara Institute of Science and Technology, 8916-5 Takayama, Ikoma, Nara 630-0192, Japan

## Abstract

Progress in the “omics” fields such as genomics, transcriptomics, proteomics, and metabolomics has engendered a need for innovative analytical techniques to derive meaningful information from the ever increasing molecular data. KNApSAcK motorcycle DB is a popular database for enzymes related to secondary metabolic pathways in plants. One of the challenges in analyses of protein sequence data in such repositories is the standard notation of sequences as strings of alphabetical characters. This has created lack of a natural underlying metric that eases amenability to computation. In view of this requirement, we applied novel integration of selected biochemical and physical attributes of amino acids derived from the amino acid index and quantified in numerical scale, to examine diversity of peptide sequences of terpenoid synthases accumulated in KNApSAcK motorcycle DB. We initially generated a reduced amino acid index table. This is a set of biochemical and physical properties obtained by random forest feature selection of important indices from the amino acid index. Principal component analysis was then applied for characterization of enzymes involved in synthesis of terpenoids. The variance explained was increased by incorporation of residue attributes for analyses.

## 1. Background

Biology and other modern sciences have become data intensive and in fact data-driven biology is now a full-fledged domain of specialization among the life sciences. Due to the amorphous nature of the accruing biological data, various databases have continually been developed to allow systematization [1]. A lot however still needs to be done to characterize these compilations into meaningful information. KNApSAcK database describes species-metabolite relationships, and within the KNApSAcK family we have developed an enzyme-reaction database called KNApSAcK motorcycle DB containing reactions and enzyme peptide sequences based on experimental evidence focusing on secondary metabolic reactions in plants.

Increasingly, the need to analyse data in such repositories has made advanced mathematical and statistical tools a mainstay of bioinformatics in recent years, more so in sequence-based analyses [2]. For molecular sequence data especially proteins, standard alphabetical notation of sequence information may not explicitly capture aspects such as their biochemical and physicochemical properties (BPPs) and may to some extent limit tractability to mathematical analyses. Ideally, computational analyses of the often heterogenous datasets require theoretical representations in forms suitable for various data processing models. This formal representation has been defined as sequence feature coding [3]. There has been no standard method of directly encoding quantifiable BPP of protein sequences hitherto. A key research question has thus been how to quantitatively characterize such data for computation whilst considering these aspects and other sequence metadata [4]. In the present study, we introduce a BPP subset for encoding amino acid residue properties into protein sequences during analyses. We found that this increases the flexibility of computational analyses focusing on facets of biochemical, physical, and evolutionary attributes of sequence data. Integration of BPP is employed in examination of diversity in enzymes related to secondary metabolite pathways, specifically those involved in terpenoid synthesis.

Researchers have proposed schemes to ensure amenability of sequences to computation, but it remains difficult to achieve computational objectivity while maintaining biological interpretability. 5-bit and 3-bit binary feature coding of amino acids in peptide sequences acids have been used in studies such as [5]. White and Seffens also used 20-bit transformation for neural network application for translation of proteins [6]. A limitation of binary feature coding is the minimal biological information with respect to amino acid diversity. This is because bit coding does not account for relative similarities or differences between amino acids and neither is it flexible to integration of their BPP; besides, binary notation of highly conserved protein sets may also pose numerical difficulties to probability-based models.

Information theory has also been exploited as an alternative, where mutual information and entropy are estimated by the Shannon-Wiener indexing of amino acid properties [7, 8]. This way, distance and variation between amino acid units, is estimable and therefore is an improvement over the binary coding method. It, however, does not directly represent characteristic attributes such as polarity, molecular size, and other features of residues.

More recently, the amino acid index (AAindex) database has accumulated published data of amino acid properties [9]. Each index has a set of 20 numerical values of a BPP quantified and published in research literature. Currently, AAindex contains 544 indices describing quantifiable amino acids residue properties. This provides a foundation for BPP feature-coding protein by assigning “scale-measured” attributes of amino acids.

AAindex in its entirety (raw form) is not merited for feature coding since it is highly redundant and has some missing values. Atchley and colleagues proposed index reduction using multivariate factor analysis reducing it to five compressed factors [10]. This methodology is a useful solution for AAindex-based metrication of amino acid residues but factor analysis (FA) reduction complicates biological interpretability in downstream analyses. This is because FA just like principal component analysis (PCA) assumes an underlying linear independence of variables whose coefficients, also called “factors,” are a proxy interpretation of AAindex variables [11]. This means that “factors” derived are pseudovariables of the actual original properties and in a way add to complexity of biological interpretation in subsequent downstream steps of sequence analyses.

In light of these challenges in analytical systematization of protein sequences, the present work applies a slightly different variable selection criterion from AAindex for the purpose of encoding BPP information into sequence data. This was achieved by use of random forest (RF) algorithm [12] to reduce redundancy and to maximize amino acid metadata captured in the AAindex. Eight BPP indices describing variability of amino acids were selected based on our experimental results. The derived reduced AAindex (rAAindex) is a subset of the original AAindex after elimination of redundancies. We further integrated the rAAindex in characterization of protein sequence diversity in the KNApSAcK motorcycle DB. The enzymes characterized are involved in secondary metabolic pathways of terpenoids and include monoterpenoid synthases, diterpenoid synthases, triterpenoid synthases, and sesquiterpenoid synthases.

## 2. Materials and Methods

### 2.1. Amino Acid Index and Random Forest Selection of Biochemical and Physical Properties

Amino acid index (AAindex) is a database of numerical indices describing biochemical and physical attributes of the 20 amino acids [9]. It provides a plausible starting point for interpreting peptide BPPs numerically through its “building blocks”: the amino acids. We selected a set of important indices that broadly characterize amino acid BPP variation, where RF algorithm [12] was used for index selection.

We denote by *X* the set of amino acid indices as the explanatory variables. *N* denotes the set of amino acids (AA). The categorical predictor variable that best defines the AA population is its qualitative attribute of water interaction; that is, every amino acid is described as either hydrophobic or hydrophilic. We therefore denote *Y* as describing hydrophobicity or hydrophilicity of an amino acid. The *i*th amino acid, *n*_*i*_, is described by a vector (*x*_1_,…, *x*_*m*_, *y*_*i*_).

Raw AAindex is highly redundant and multicollinear. We initially processed it by removal of indices that had missing values for any amino acid. Redundant indices were eliminated by backward elimination of variables whose correlation coefficient was above a threshold of 0.85. 283 indices were retained for RF variable selection.

Random forest (RF) [12] is a popular algorithm in statistics and bioinformatics for two reasons:

- it is a powerful classification and regression tree (CART) tool that generates ensembles of decision trees. RF and other decision tree-based classifiers are nonparametric; that is, they do not assume underlying structure in a dataset and are useful for classification and regression modeling of complex biological data;
- RF implements a mechanism of calculating variable importance scores (VIM) by permutation testing. These measures are useful in feature selection and provide an advantage which we explore in this work.

**Figure 1:**
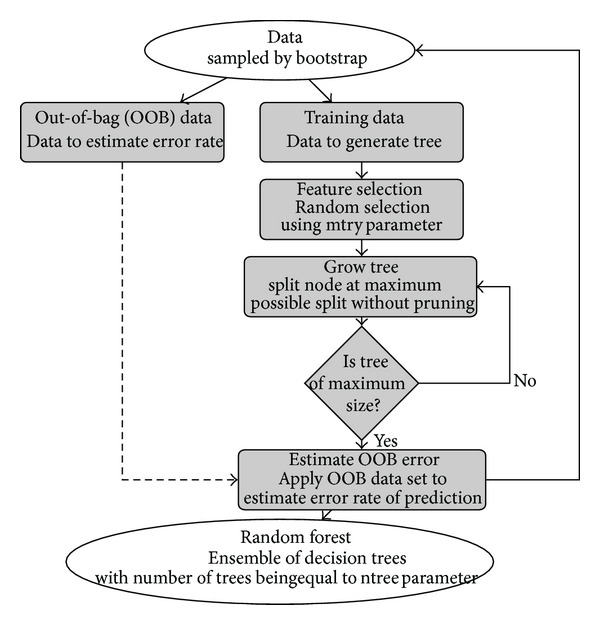
Random forest algorithm. Mechanism of the random forest (RF) algorithm starting from the data selection by bootstrapping up to variable importance calculation. The amino acid index data containing physicochemical metrics of amino acids was subjected to RF for index selection.

Besides these two advantages, further application to biological research has been documented in [14]. Detailed mechanism of the RF algorithm is described in [12, 15] although a generalized outlook of its concepts is illustrated in Figure 1. RF was implemented for selection of reduced AAindex (rAAindex) consisting of indices describing BPPs explaining the variation of amino acids.

In RF, sampling by bootstrap creates an “out-of-bag” (OOB) sample which is an important feature due to its usefulness in estimation of VIM. These scores are derived by permutation testing of the OOB data in the error estimation step. VIM scores of index *x*_*i*_ are described as the mean error rate over all trees in the RF ensemble. Detailed information on VIM calculation is described elsewhere [12], but for descriptive purposes, we simplify the formal representation of this measure as

(1) $$\begin{array}{c} \text{VIM}\left(x_{i}\right)=\frac{1}{\text{ntree}}\overset{\text{ntree}}{\underset{1}{\sum }}\left(\tilde{\mathrm{err}\cdot \text{OO}{\text{B}}_{\text{t}}}-\mathrm{err}\cdot \text{OO}{\text{B}}_{\text{t}}\right), \end{array}$$

where VIM(*x*_*i*_) is a function estimating the VIM score for variable *x*_*i*_ and ntree is the number of trees in the RF ensemble, whereas $\tilde{\mathrm{err}\cdot \text{OO}{\text{B}}_{\text{t}}}$ is the number of misclassifications tested on a tree t where the input was permuted values of variable *x*_*i*_. Conversely, *err*⁡·OOB_t_ is the number of misclassifications tested on a tree t whose input was the nonpermuted values of variable *x*_*i*_.

Validity of permutation test derivation of VIM in the RF algorithm operates on the premise that if a variable is “important,” then permuting its values (realistically) leads to reduced accuracy of class prediction. Variables were selected using the method described in [16]. The first step involved stochastically running 1000 RF classification trials of AAindex and each time recording the mean decrease in accuracy (VIM score). Indices were then ranked on a decreasing score order. The variation of these VIM scores was obtained and the point of minimum variance was initialized as a threshold, from which 93 amino acid indices were retained for the further index reduction by nested RF feature selection approach described in detail by Genuer et al. [16]. The threshold of significant deviation in the increasing error rates from the nested RF modeling was set to the number of variables above which the error rates significantly increase above the threshold of 0.02 percent meaning that at most a single amino acid misclassification could be accepted from the nested RF. A reduced amino acid index (rAAindex) was thus derived and its usefulness as a representation of amino acid information was tested on data from our KNApSAcK motorcycle database.

### 2.2. KNApSAcK Motorcycle DB: Peptide Sequence-Metabolic Reaction Relationship DB

**Figure 2:**
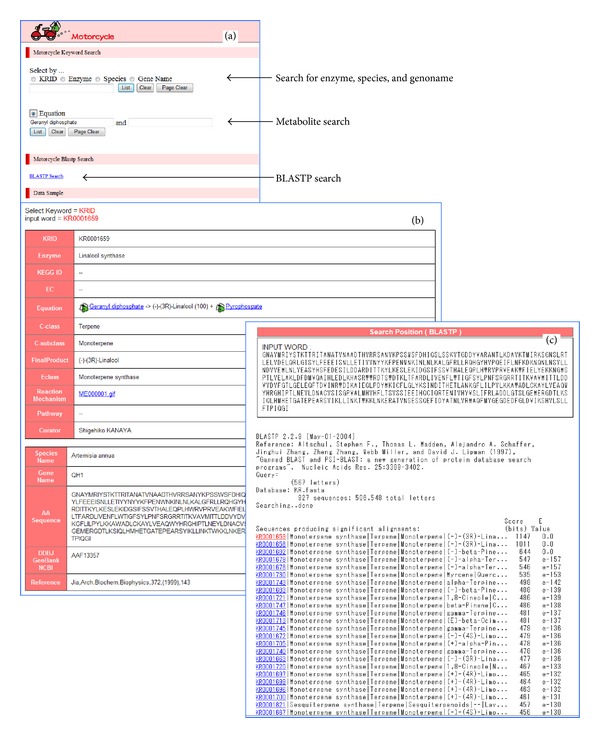
KNApSAcK motorcycle database. Enzyme-reaction database. (a) The main window of motorcycle. (b) An example of a keyword search. (c) An example of BLASTP search.

It is necessary to extend the species-metabolite relationship DB by incorporating a secondary metabolite pathway DB that includes pathways with detected enzymatic reactions and other actual or predicted peptide sequences that may be involved in these pathways. We surveyed reactions of secondary metabolites in scientific literature, and amino acid sequences involved in secondary metabolism were obtained from public databases in PubMed.gov (http://www.ncbi.nlm.nih.gov/). All the data comprising 2,881 secondary metabolic reactions was accumulated in the KNApSAcK motorcycle DB (http://kanaya.naist.jp/motorcycle/top2.html) as shown in the main window of the KNApSAcK motorcycle DB (Figure 2); enzyme reactions can be retrieved using keywords of enzymes, species, genes, metabolites, and peptide sequences obtained from a BLASTP search. For metabolite search using its keywords, we obtain information on enzyme name, reaction involved, compound class (C-class in Figure 2) and subclass (C-subclass) of metabolic reactions, and reaction mechanisms. For BLASTP search, we can predict reaction equations for a targeted peptide sequence using information on the class and subclass of metabolic pathways (Figure 1(c)). Thus, the motorcycle DB makes it possible to predict enzyme reactions based on the class and subclass of metabolic reactions evidenced by experiments mentioned in scientific literature. This differentiates it from KEGG [17] and BioCyc [18]. We have thus far obtained 596,974 protein sequences of 59,165 plant species and 124,292 protein sequences of 66 bacterial species from the nonredundant protein sequences of PlantGDB.

For analytical purposes of the presently developed method, we narrowed our test dataset to terpenoid synthase peptide sequences with >200 amino acid residues. Terpenoids are organic metabolites of plants that have been shown to have insect-pesticide properties among other roles. Terpenoid synthases sequence from the KNApSAcK database [19] was examined for patterns in diversity. Enzymes annotated to four families, namely, monoterpenoid synthases, diterpenoid synthases, triterpenoid synthases, and sesquiterpenoid synthases, were examined by PCA. Understanding the data structure of these terpenoid enzyme subfamilies is important for annotation of similar organic compounds [20]. Multiple sequence alignment and gap removal were carried out to extract homologous regions of sequences from the four subfamilies of terpenoid synthases. These domains had a length of 28 residues for the 283 sequences. Binary and rAAindex (BPP) feature coding of amino acid residues in these sequences were compared.

### 2.3. Sequence Diversity Characterization Based on Principal Component Analyses (PCA)

The present work attempts to examine diversity of secondary metabolic enzyme groups using datasets from the KNApSAcK motorcycle DB by integrating amino acid attributes represented in the rAAindex where PCA was used to analyse variation. PCA is a technique that enables efficient interpretation of variation and relationship between variables in a huge dataset represented by higher dimensional vectors [21]. It is widely applied in bioinformatics as exemplified by Tatusov et al. who phylogenetically classified genomes by protein function [22]. For comparative purposes, a BPP integrated dataset was analysed in comparison to 8-bit binary-coded enzyme set. We initially generated lattices representing individual sequences and encoded by both rAAindex and the commonly used 8-bit binary feature coding. We hypothesized that the BPP-encoded set explains more variance of sequences and thus reflects the diversity of proteins based on BPP integration.

## 3. Results and Discussion

### 3.1. Reduced Amino Acid Index

Physicochemical properties of amino acids quantitatively describe the overall biochemical behaviour of peptide and protein sequences [13]. The amino acid index database [9] has collected properties of amino acids measured by various researchers since the 1970s using scientific instruments and quantifiable metrics. It is essential to consider these properties in objective analyses of sequence data. Numeric quantification is also pivotal because it gives a flexible way of integrating this information in a mathematically and statistically amenable form different from the alphabetical string representation.

The AAindex database is highly redundant and has some missing values for certain properties. In its raw form, the AAindex is unsuitable for direct BPP encoding. Ideally, a reduced set would work for most sequence analyses. Researchers have utilized various compression techniques to reduce the AAindex. Atchley and colleagues used a multivariate factor analysis to propose a compressed variable set of five vectors describing amino acids in a multidimensional space [10]. More recently, fuzzy c-means algorithm has been applied in clustering the AAindex indices and the resultant clusters incorporated in a support vector machine modeling experiment to predict DNA-binding domains [23].

Factor analysis (FA) is a useful approach for the same purpose of defining a minimal set of “factors” that simplify interpretation of protein sequence characteristics. Random forest (RF) variable reduction differs from FA since RF selects important variables without compressing the whole variable set into fewer descriptive factors. RF has been proven to be a useful tool for biological data as described in [24–26]. Here, BPP selection entails minimizing the original variables in the AAindex by elimination of redundancy, high collinearity, and less informative variables whilst maintaining a sufficiently parsimonious set of the original BPP properties represented in the AAindex. Compression (as in FA) on the other hand is redefinition of original AAindex variables into new components by multivariate techniques such as PCA [21] and factor analyses [27]. We argue against compression in the context of AAindex data that while a minimized descriptive set is achieved, there results a complexity of biological interpretation that arises if the redefined variables are applied in subsequent downstream mathematical or statistical analyses.

**Figure 3:**
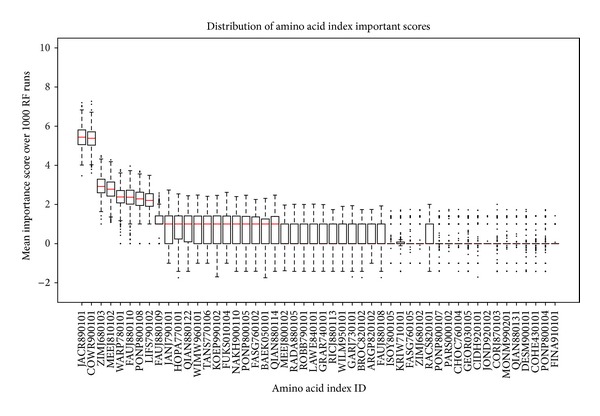
Variable importance scores (1000 RF trials). Variable importance score distribution for 1000 runs of random forest classification on the amino acid index. Each boxplot represents distribution of each property (also called variable) represented on the horizontal axis. The properties have been ordered in decreasing order of the median score (red line in boxplot). For easier visualization, the set has been truncated to show the top 50 properties. The corresponding properties are shown in Table 1.

**Figure 4:**
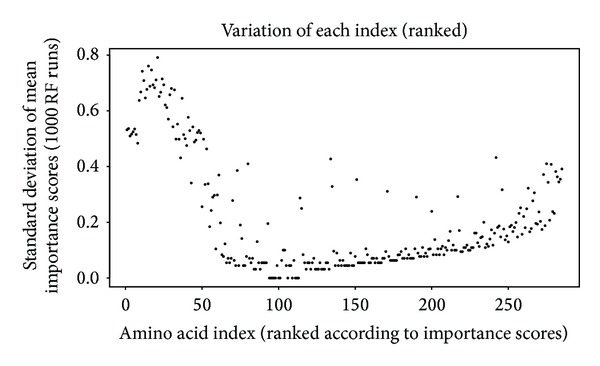
Variation of the BPP importance. Standard deviation of the importance scores of the properties (*y*-axis); models contribution of each property to the performance of the RF algorithm. Those variables with a close to zero variation are less “important.” At the tail end, variance is more than zero due to chance.

**Figure 5:**
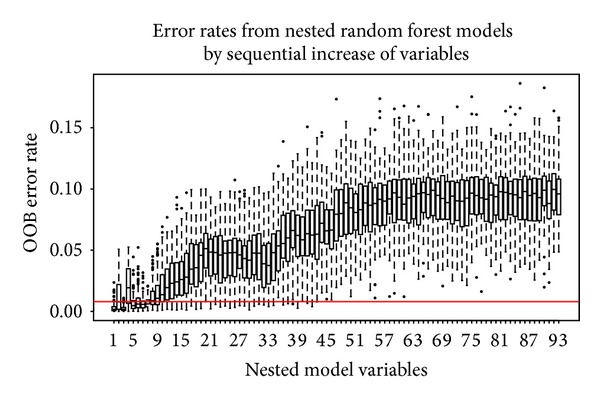
Nested random forest error rates. Nested random forest variable selection: variables have been ordered by their importance scores, new RF models are built by single variable addition in the nested RF setup, and RF error rates are measured. In this experiment, each box and whisker plot represents the distribution of error rates from 100 trials at each nested RF step. In total, there were 93 steps corresponding to the 93 top indices from previous step. The *y*-axis shows the error rates as a percentage. The threshold of acceptable mean error rate was set at 2 percent shown by the red horizontal line.

From the initial 544 properties contained in the amino acid index database, 13 which had missing values were dropped. Initially, the redundancy was further reduced by dropping variables with a correlation coefficient greater than 0.85, further trimming the set to 283 amino acid indices. The retained indices were then subjected to the RF algorithm (1000 trials) and variable reduction was done using the technique in [16]. Variable importance scores (VIM) were ranked in decreasing order (Figure 3). The “importance” score of a BPP illustrates its significance with regard to amino acid classification. It shows the VIM score distribution for 1000 runs of random forest classification of BPP in the amino acid index. Each boxplot in the figure represents distribution of each BPP (also called variable) represented on the horizontal axis. These properties have been ordered in decreasing order of the median score (red line in boxplot). For easier visualization, the set in the figure has been truncated to show the top 50 properties by mean VIM ranking. The corresponding properties are shown in Table 1. Variation (standard deviation) of the ranked scores was observed as shown in Figure 4. Standard deviation of the importance scores of the properties (*y*-axis) models contribution of each property towards performance of the RF algorithm. Those variables with a close to zero variation are less “important.” At the tail end variance is higher than zero but is large due to chance (*P* value > 0.05). Variables with a mean VIM score of 0 or less were dropped, lowering the retained variables to 93 indices. Nested RF modeling was done using the ranked indices, by starting with the highest ranked variable and subsequent addition remaining after each step. Error rates were estimated for each step of the nested modeling. Details of nested RF are explained in [16]. The threshold of acceptable error rate was set to 2 percent.  Figure 5 shows that when the first 8 indices are used for classification, the error rate remains under the 2 percent error rate (horizontal red line in the figure) threshold, whereas it significantly rises with the addition of subsequent indices.

An RF-reduced subset of the amino acid index, rAAindex, (Table 2) with these 8 most important BPPs, is proposed for use in BPP encoding especially for statistical learning and other mathematical tasks involving protein sequences. The properties retained are shown in Table 3.

### 3.2. Characterization of Terpene Synthase Sequence Diversity

**Figure 6:**
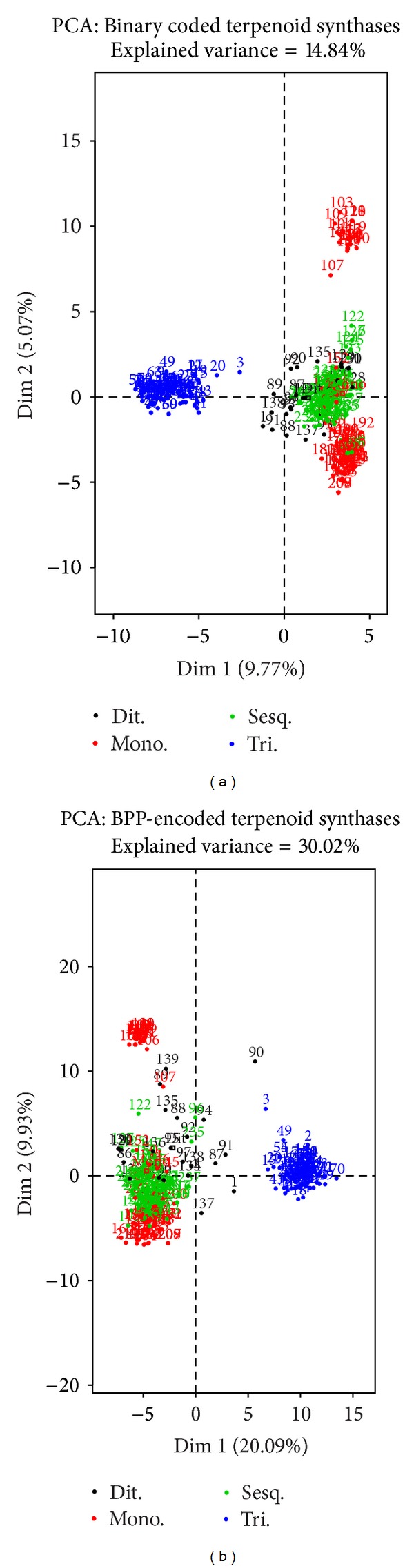
Principal component analysis of terpenoid synthases. Left: principal component analysis of the terpenoid synthase subfamilies where amino acid residues are encoded in 8-bit binary method. PC1 and PC2 show a combined variance of 14.84 percent explained variance. Right: principal component analysis of the same data set encoded using the biochemical and physical properties of amino acid residues. PC1 and PC2 in this case explain 30.02 percent variance. The four subfamilies clustered are monoterpenoid, diterpenoid, triterpenoid, and sesquiterpenoid synthases. Triterpenoid synthases and subgroups of terpenoid synthases are distinctly different in structure compared to the other synthases.

Terpenes are the largest group of plant natural products with a variety of core chemical structures comprising at least 30,000 compounds and synthesized by terpenoid synthases [28]. Terpene diversity is caused by the large number of different terpene synthases used in the first step of terpene synthesis, and some terpene synthases produce multiple products [29]. Terpene synthases are generally classified according to the number of carbons in their substrates, that is, geranyl diphosphate (C10, GPP) for monoterpene synthases, farnesyl diphosphate (C15, FPP) for sesquiterpene synthases, geranylgeranyl diphosphate (C20, GGPP) for diterpene synthases, and squalene for triterpene synthases (C30). The rather limited similarity of plant terpenoids [20] complicates annotation of their enzymes. Clustering algorithms improve the resolution to some extent. PCA was used to analyse variation among 4 terpene synthase subfamilies. We examined the performance of PCA classification when rAAindex BPP encoding is implemented, relative to the commonly used 8-bit binary encoding. The combined variance explained by the first two principal components is 30.02 (Figure 6 left) percent, whereas the variance explained by the first two components in binary encoded set is 14.84 percent (Figure 6 right). Triterpenoid synthases can be clearly distinguished from the other categories, which was also consistent with our previous findings [30].

We compared the performance of rAAindex to 8-bit binary encoding of amino acids [31] in an actual dataset (described in the data and methods section). Binary coding is the most popular representation scheme for machine learning tasks of protein data [5, 6] and was utilized as a benchmark of comparison to rAAindex encoding. Figure 6 (left) shows the fragment distribution of terpene synthases coded by 8-bit binary notation mainly clustered into five regions. Similarly, Figure 6 (right) shows the distribution of terpene synthases coded by rAAindex into the same 5 groups. Principal component analysis was performed on terpenoid synthase sequence subfamilies, where amino acid residues were encoded in 8-bit binary code. PC1 and PC2 show a combined variance of 14.84 percent explained variance. The second part of the figure illustrates PCA of the same data set encoding the biochemical and physical properties of amino acid residues described above. PC1 and PC2 in this case explain 30.02 percent variance showing that more sequence information is described by the BPP subset. The four subfamilies clustered are monoterpenoid, diterpenoid, triterpenoid, and sesquiterpenoid synthases. Triterpenoid synthases and subgroups of terpenoid synthases are distinctly different in structure compared to the other synthases. It is noted that three types of terpene synthases except for the diterpene synthases are less divergent at the peptide sequence level; that is, small changes in peptide sequences of the terpene synthase make it possible to synthesize many different terpenoid compounds. The orders of fragments from the N- to the C-terminus in enzymes are arranged in two clusters for monoterpene synthases and are a single cluster for the other categories. Thus, monoterpene and sesquiterpene synthases are very similar in arrangement of peptide fragments, which is consistent with the similarity of the 3D structures in monoterpene and sesquiterpene synthases [32, 33] and with the fact that several bifunctional enzymes possess both sesquiterpene and monoterpene synthase activities [33, 34].

Diterpene and triterpene synthases have inherent structures specified by a single cluster for diterpene synthases and two clusters from the N- to C-terminus for triterpene synthases.

## 4. Conclusion

This paper has introduced a subset of eight biochemical and physical attributes of amino acids that can be encoded in protein sequences for sequence-based analysis. These features quantify attributes of individual residues in numerical metrics that improve amenability to mathematical and statistical tasks and also enhance biological interpretability of such tasks. Terpenoid synthases protein set was used to evaluate the encoding of these attributes by PCA. The terpenoid synthase subfamilies established that more variance is explained when BPPs are encoded compared to the commonly used binary encoding which does not integrate physicochemical aspects of protein sequences.

## Supplementary Material

The supplementary figure 1 depicts the procedure carried out on this report. The starting point was the amino acid index database, which was reduced to a small subset based on variable importance scores (VIM) derived by random forest feature selection. The reduced set (rAAindex) was further used to encode biochemical and physical properties into protein sequences and subsequently for examination of the data structure of the sub-families terpene synthase sequences.
Also included with the supplementary materials are: the amino acid index, the list of 544 properties in the amino acid index and a short documentation of the amino acid index data and format.

## Figures and Tables

**Table 1:** Top 50 ranked indices. Descriptions of the top 50 indices by ranking of the VIM scores prior to nested RF. The first column represents the AAindex access ID, while the second is the corresponding BPP. Please refer to the amino acid index database in the Supplementary Material available online at http://dx.doi.org/10.1155/2014/753428 for the references in column 2.

	ID	Property
1	RADA880101	Information value for accessibility; average fraction 35% (Biou et al., 1988)
2	ROSM880101	Information value for accessibility; average fraction 23% (Biou et al., 1988)
3	KIDA850101	Retention coefficient in TFA (Browne et al., 1982)
4	EISD840101	Normalized hydrophobicity scales for alpha + beta-proteins (Cid et al., 1992)
5	JACR890101	Normalized hydrophobicity scales for alpha/beta-proteins (Cid et al., 1992)
6	COWR900101	Consensus normalized hydrophobicity scale (Eisenberg, 1984)
7	BLAS910101	Direction of hydrophobic moment (Eisenberg-McLachlan, 1986)
8	MEEJ810101	Hydrophobic parameter pi (Fauchere-Pliska, 1983)
9	CIDH920104	Number of hydrogen bond donors (Fauchere et al., 1988)
10	GRAR740102	Number of full nonbonding orbitals (Fauchere et al., 1988)
11	ZIMJ680103	Polarity (Grantham, 1974) [13]
12	MEEJ810102	Hydration number (Hopfinger, 1971), Cited by Charton-Charton (1982)
13	RADA880104	Hydropathy index (Kyte-Doolittle, 1982)
14	KUHL950101	Hydrophobic parameter (Levitt, 1976)
15	FAUJ880110	Conformational preference for parallel beta-strands (Lifson-Sander, 1979)
16	RADA880107	Average surrounding hydrophobicity (Manavalan-Ponnuswamy, 1978)
17	WARP780101	Retention coefficient in NaClO4 (Meek-Rossetti, 1981)
18	BIOV880102	Retention coefficient in NaH2PO4 (Meek-Rossetti, 1981)
19	BIOV880101	8 A contact number (Nishikawa-Ooi, 1980)
20	FASG890101	Partition coefficient (Pliska et al., 1981)
21	PONP800108	Average number of surrounding residues (Ponnuswamy et al., 1980)
22	FAUJ830101	Hydrophobicity (Prabhakaran, 1990)
23	CORJ870101	Weights for beta-sheet at the window position of 2 (Qian-Sejnowski, 1988)
24	MANP780101	Transfer-free energy from chx to wat (Radzicka-Wolfenden, 1988)
25	LIFS790102	Transfer-free energy from chx to oct (Radzicka-Wolfenden, 1988)
26	GUOD860101	Energy transfer from out to in (95% buried) (Radzicka-Wolfenden, 1988)
27	WOLS870101	Mean polarity (Radzicka-Wolfenden, 1988)
28	WOLR790101	Side chain hydropathy, uncorrected for solvation (Roseman, 1988)
29	LEVM760101	Side chain hydropathy, corrected for solvation (Roseman, 1988)
30	WOLR810101	Normalized frequency of chain reversal D (Tanaka-Scheraga, 1977)
31	ROSM880102	Average interactions per side chain atom (Warme-Morgan, 1978)
32	WOEC730101	Polar requirement (Woese, 1973)
33	PLIV810101	Hydration potential (Wolfenden et al., 1981)
34	RADA880108	Principal property value z1 (Wold et al., 1987)
35	FAUJ880109	Polarity (Zimmerman et al., 1968)
36	HOPA770101	Free energies of transfer of AcWl-X-LL peptides from bilayer interface to water (Wimley-White, 1996)
37	CIDH920103	Hydropathy scale based on self-information values in the two-state model (9% accessibility) (Naderi-Manesh et al., 2001)
38	TANS770106	Hydropathy scale based on self-information values in the two-state model (16% accessibility) (Naderi-Manesh et al., 2001)
39	PRAM900101	Hydrophilicity scale (Kuhn et al., 1995)
40	ENGD860101	Retention coefficient at pH 2 (Guo et al., 1986)
41	BROC820101	Modified Kyte-Doolittle hydrophobicity scale (Juretic et al., 1998)
42	NADH010103	Knowledge-based membrane-propensity scale from 1D_Helix in MPtopo databases (Punta-Maritan, 2003)
43	NADH010102	Hydrophobicity index (Wolfenden et al., 1979)
44	KYTJ820101	Hydrophobicity-related index (Kidera et al., 1985)
45	EISD860103	Weights from the IFH scale (Jacobs-White, 1989)
46	NISK800101	Hydrophobicity index, 3.0 pH (Cowan-Whittaker, 1990)
47	JURD980101	Scaled side chain hydrophobicity values (Black-Mould, 1991)
48	WIMW960101	NNEIG index (Cornette et al., 1987)
49	QIAN880122	Hydrophobicity index (Engelman et al., 1986)
50	PUNT030101	Hydrophobicity index (Fasman, 1989)

**Table 2:** Reduced Amino Acid Index (rAAindex). The first column contains the names of the 20 amino acids that make up protein sequences. Each of the 8 subsequent columns is a BPP attribute selected based on its importance in explaining variation of the amino acid. Each row is a vector describing an amino acid in 8 dimensions, each of which represents a physical property tabulated in Table 3.

Amino acid	JACR890101	COWR900101	ZIMJ680103	MEEJ810102	FAUJ880110	WARP780101	PONP800108	LIFS790102
Ala	0.18	0.42	0.00	1.00	0.00	10.04	6.05	1.00
Arg	−5.40	−1.56	52.00	−2.00	3.00	6.18	5.70	0.68
Asn	−1.30	−1.03	3.38	−3.00	3.00	5.63	5.04	0.54
Asp	−2.36	−0.51	49.70	−0.50	4.00	5.76	4.95	0.50
Cys	0.27	0.84	1.48	4.60	0.00	8.89	7.86	0.91
Gln	−1.22	−0.96	3.53	−2.00	3.00	5.41	5.45	0.28
Glu	−2.10	−0.37	49.90	1.10	4.00	5.37	5.10	0.59
Gly	0.09	0.00	0.00	0.20	0.00	7.99	6.16	0.79
His	−1.48	−2.28	51.60	−2.20	1.00	7.49	5.80	0.38
Ile	0.37	1.81	0.13	7.00	0.00	8.72	7.51	2.60
Leu	0.41	1.80	0.13	9.60	0.00	8.79	7.37	1.42
Lys	−2.53	−2.03	49.50	−3.00	1.00	4.40	4.88	0.59
Met	0.44	1.18	1.43	4.00	0.00	9.15	6.39	1.49
Phe	0.50	1.74	0.35	12.60	0.00	7.98	6.62	1.30
Pro	−0.20	0.86	1.58	3.10	0.00	7.79	5.65	0.35
Ser	−0.40	−0.64	1.67	−2.90	2.00	7.08	5.53	0.70
Thr	−0.34	−0.26	1.66	−0.60	2.00	7.00	5.81	0.59
Trp	−0.01	1.46	2.10	15.10	0.00	8.07	6.98	0.89
Tyr	−0.08	0.51	1.61	6.70	2.00	6.90	6.73	1.08
Val	0.32	1.34	0.13	4.60	0.00	8.88	7.62	2.63

**Table 3:** Annotation of the selected properties. Descriptions of the 8 indices selected after the nested random forest variable selection. The first column represents the AAindex access ID, while the second is the corresponding BPP. Please refer to the amino acid index database for the references in column 2.

	ID	Property
1	JACR890101	Number of full nonbonding orbitals (Fauchere et al., 1988)
2	COWR900101	Conformational preference for parallel beta-strands (Lifson-Sander, 1979)
3	ZIMJ680103	Retention coefficient in NaH2PO4 (Meek-Rossetti, 1981)
4	MEEJ810102	Average number of surrounding residues (Ponnuswamy et al., 1980)
5	WARP780101	Average interactions per side chain atom (Warme-Morgan, 1978)
6	FAUJ880110	Polarity (Zimmerman et al., 1968)
7	PONP800108	Weights from the IFH scale (Jacobs-White, 1989)
8	LIFS790102	Hydrophobicity index, 3.0 pH (Cowan-Whittaker, 1990)
