# Supplementary material for: Integration of Residue Attributes for Sequence Diversity Characterization of Terpenoid Enzymes
Source: Biomed Res Int. 2014 May 11;2014:753428. doi: 10.1155/2014/753428 (PMC4034505; doi:10.1155/2014/753428)
Supplement: Supplementary file 1 — The supplementary figure 1 depicts the procedure carried out on this report. The starting point was the amino acid index database, which was reduced to a small subset based on variable importance scores (VIM) derived by random forest feature selection. The reduced set (rAAindex) was further used to encode biochemical and physical properties into protein sequences and subsequently for examination of the data structure of the sub-families terpene synthase sequences. Also included with the supplementary materials are: the amino acid index, the list of 544 properties in the amino acid index and a short documentation of the amino acid index data and format. [file 753428.f1.zip › mat.753428.v1.pdf]

Additional file 1

Each entry in the amino acid index has information organized as:
H Accession number
D Data description
R LITDB entry number
A Author(s)
T Title of the article
J Journal reference
C Accession numbers of similar entries with the correlation coefficients of 0.8 (-0.8) or more (less).
I Amino acid index data in the following order

Ala	Arg	Asn	Asp	Cys	Gln	Glu	Gly	His	Ile
Leu	Lys	Met	Phe	Pro	Ser	Thr	Trp	Tyr	Val

// Index separator symbol

An example amino acid index is shown below.

```
H  ANDN920101
D  alpha-CH chemical shifts (Andersen et al., 1992)
R  LIT:1810048b PMID:1575719
A  Andersen, N.H., Cao, B. and Chen, C.
T  Peptide/protein structure analysis using the chemical shift index
method:  upfield alpha-CH values reveal dynamic helices and aL sites
J  Biochem.  and Biophys.  Res.  Comm.  184, 1008-1014 (1992)
C  BUNA790102 0.949

 I  A/L   R/K   N/M   D/F   C/P   Q/S   E/T   G/W   H/Y   I/V
    4.35  4.38  4.75  4.76  4.65  4.37  4.29  3.97  4.63  3.95
    4.17  4.36  4.52  4.66  4.44  4.50  4.35  4.70  4.60  3.95

//
```

Figure 1: A sample entry in the AAindex database
