# Supplementary material for: Integration of Residue Attributes for Sequence Diversity Characterization of Terpenoid Enzymes
Source: Biomed Res Int. 2014 May 11;2014:753428. doi: 10.1155/2014/753428 (PMC4034505; doi:10.1155/2014/753428)
Supplement: Supplementary file 1 — The supplementary figure 1 depicts the procedure carried out on this report. The starting point was the amino acid index database, which was reduced to a small subset based on variable importance scores (VIM) derived by random forest feature selection. The reduced set (rAAindex) was further used to encode biochemical and physical properties into protein sequences and subsequently for examination of the data structure of the sub-families terpene synthase sequences. Also included with the supplementary materials are: the amino acid index, the list of 544 properties in the amino acid index and a short documentation of the amino acid index data and format. [file 753428.f1.zip › mat.753428.v2.pdf]

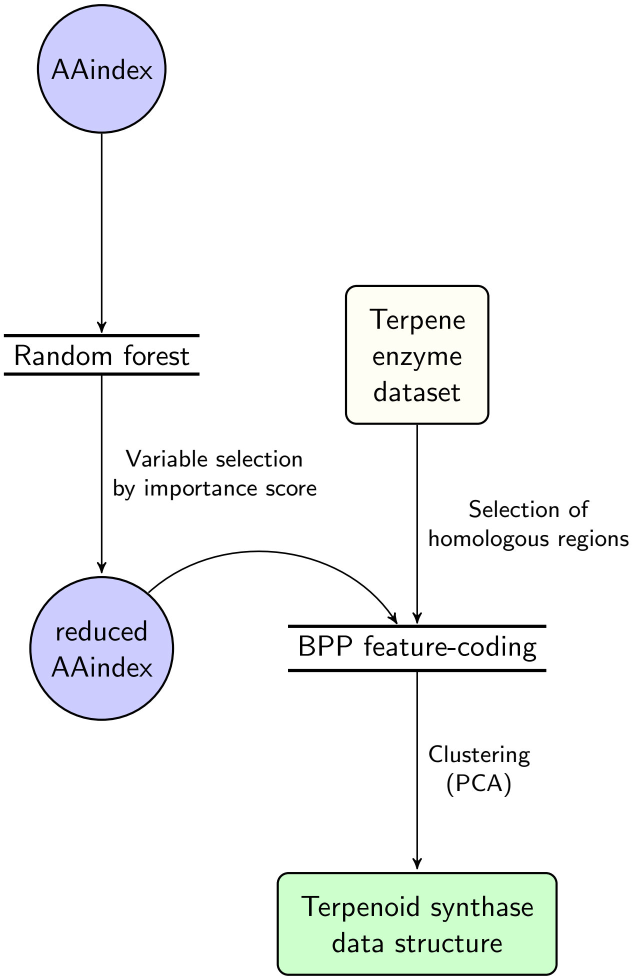

Figure 1: Materials and methods: depicts the procedure carried out on this report. The starting point is the amino acid index database which is reduced to a small subset based on variable importance scores derived from random forest algorithm. The reduced set (rAAindex) is used for to encode biochemical and physical properties into protein sequences for examination of the data structure of the sub-families terpene synthases.
